# Characterization of the complete chloroplast genome of *Phragmites australis* as a Chinese herb from Phragmites and Poaceae

**DOI:** 10.1080/23802359.2020.1720544

**Published:** 2020-01-31

**Authors:** Huaichong Wang, Xiaoxuan Chen, Tianyi Cao, Qian Ji

**Affiliations:** a Department of Pharmacy, Integrated Chinese and Western Medicine Hospital of Zhejiang Province, Hangzhou, Zhejiang, China;; b Shandong University of Traditional Chinese Medicine, Jinan, Shandong, China;; c Zhejiang Integrated Traditional Chinese and Western Medicine Hospital, Hangzhou, Zhejiang, China;; d Department of Pharmacy, Xixi Hospital of Hangzhou, Hangzhou, Zhejiang, China

**Keywords:** *Phragmites australis*, Phragmites, Poaceae, phylogenetic relationship, chloroplast

## Abstract

The root of *Phragmites australis* can often be used as the Chinese herb in China. In this paper, the complete chloroplast genome of *P. australis* was studied for more genetic information. The chloroplast genome was 137,561 bp in length as the circular and typical quadripartite structure, which contained a large single-copy region (LSC) of 82,328 bp, a small single-copy region (SSC) of 12,699 bp, and a pair of inverted-repeat regions (IRs) of 21,267 bp. The overall nucleotide composition of chloroplast genome is: 42,300 bp A (30.7%), 42,090 bp T (30.6%), 26,500 bp C (19.3%), 26,671 bp G (19.4%) and the total G + C content of 38.7%. However, 134 genes were found that included 87 protein-coding genes (PCGs), 39 transfer RNA (tRNAs), and 8 ribosome RNA (rRNAs). The Maximum-Likelihood (ML) method phylogenetic relationship with the reported chloroplast genomes showed that *Phragmites australis* is closely related to *Setaria viridis* and *Setaria italic* of the family Poaceae.

The root of *Phragmites australis* can often be used as the perennial Chinese herb in China, which belongs to the genus Phragmites. The root of *P. australis* is antiasthmatic, antiemetic, antipyretic, antitussive, depurative, diuretic, febrifuge, lithontriptic, sedative, sialogogue, and stomachic. It can be taken internally in the treatment of diarrhea, fevers, vomiting, and coughs with thick dark phlegm, lung abscesses, urinary tract infections, and food poisoning (especially from seafoods). Externally, it is mixed with gypsum and used to treat halitosis and toothache. The root is harvested in the autumn as the Chinese herb is juiced or dried for use in decoctions in China (Packer et al. 2017). The root of *P. australis* is very important for people to treat a complaint, but less information of *P. australis* about genome data and study on the evolution of this species. In this paper, we have studied the chloroplast genome of *P. australis* that can help to study the relationship between origin and evolution, also can be used to provide basic data and information of the genus Phragmites species for the development of Chinese herb for people in the future.

For this study, we used the Plant Tissues Genomic DNA Extraction Kit (TIANGEN, BJ, and CN), the total genomic DNA was isolated from the fresh roots of *Phragmites australis* and collected from herb market near Zhejiang Chinese Medical University that is located at Hangzhou, Zhejiang, China (30.09 N, 119.89E). The chloroplast genome DNA was stored in Zhejiang Chinese Medical University (No. SCMC-ZJU-TCM-09). Then, the DNA was purified and sequenced and the collected raw sequences were quality controlled and removed by the FastQC (Andrews 2015). The chloroplast genome of *P. australis* was assembled and annotated by the MitoZ (Meng et al. 2019). The chloroplast genome map was generated by the OrganellarGenomeDRAW (Lohse et al. 2013). The annotated chloroplast genome sequence of *P. australis* was from the GenBank that the accession was No. KF730315.1.

The complete chloroplast genome sequence of *P. australis* was 137,561 base pairs (bp) long and had a typical quadripartite structure, which contained a large single-copy region (LSC) of 82,328 bp, a small single-copy region (SSC) of 12,699 bp, and a pair of inverted repeat regions (IRs) of 21,267 bp in each one. The overall nucleotide composition of chloroplast genome is: 42,300 bp A (30.7%), 42,090 bp T (30.6%), 26,500 bp C (19.3%), 26,671 bp G (19.4%), and the total G + C content of 38.7%. The chloroplast genome of *P. australis* contains 134 genes, which includes 87 protein-coding genes (PCG), 39 transfer RNA genes (tRNAs), and 8 ribosomal RNA genes (rRNAs). In addition, 21 genes were found duplicated in IR regions, which included 9 PCGs species (*rps19, rpl2, rpl23, ndhB, rps7,* two of *rps12, ycf68, a*nd *rps15*), 8 tRNAs species (*trnH-GUG, trnI-CAU, trnL-CAA, trnV-GAC, trnI-GAU, trnA-UGC, trnR-ACG,* and *trnN-GUU*), and 4 rRNAs species (*rRNA16, rRNA23, rRNA4.5,* and *rRNA5*).

To study the phylogenetic relationship of *P. australis* with other 18 species chloroplast genomes that we used the Maximum-Likelihood (ML) method to construct the phylogenetic tree. The phylogenetic tree of ML analysis was performed using the MEGA X (Kumar et al. 2018) with best model and all of the nodes were inferred with strong support and used the bootstrap values from 1000 replicates. The phylogenetic tree was drawn using the MEGA X and edited using the Evolview web (www.evolgenius.info/evolview) (Subramanian et al. 2019). The Maximum-Likelihood (ML) method phylogenetic relationship with the reported chloroplast genomes showed that *Phragmites australis* is closely related to *Setaria viridis* (NC028075.1) and *Setaria italic* (KJ001642.1) in family Poaceae (Figure 1). This study can be used to provide basic data and information of the genus Phragmites species for the development of Chinese herb for people in the future.

**Figure 1.**
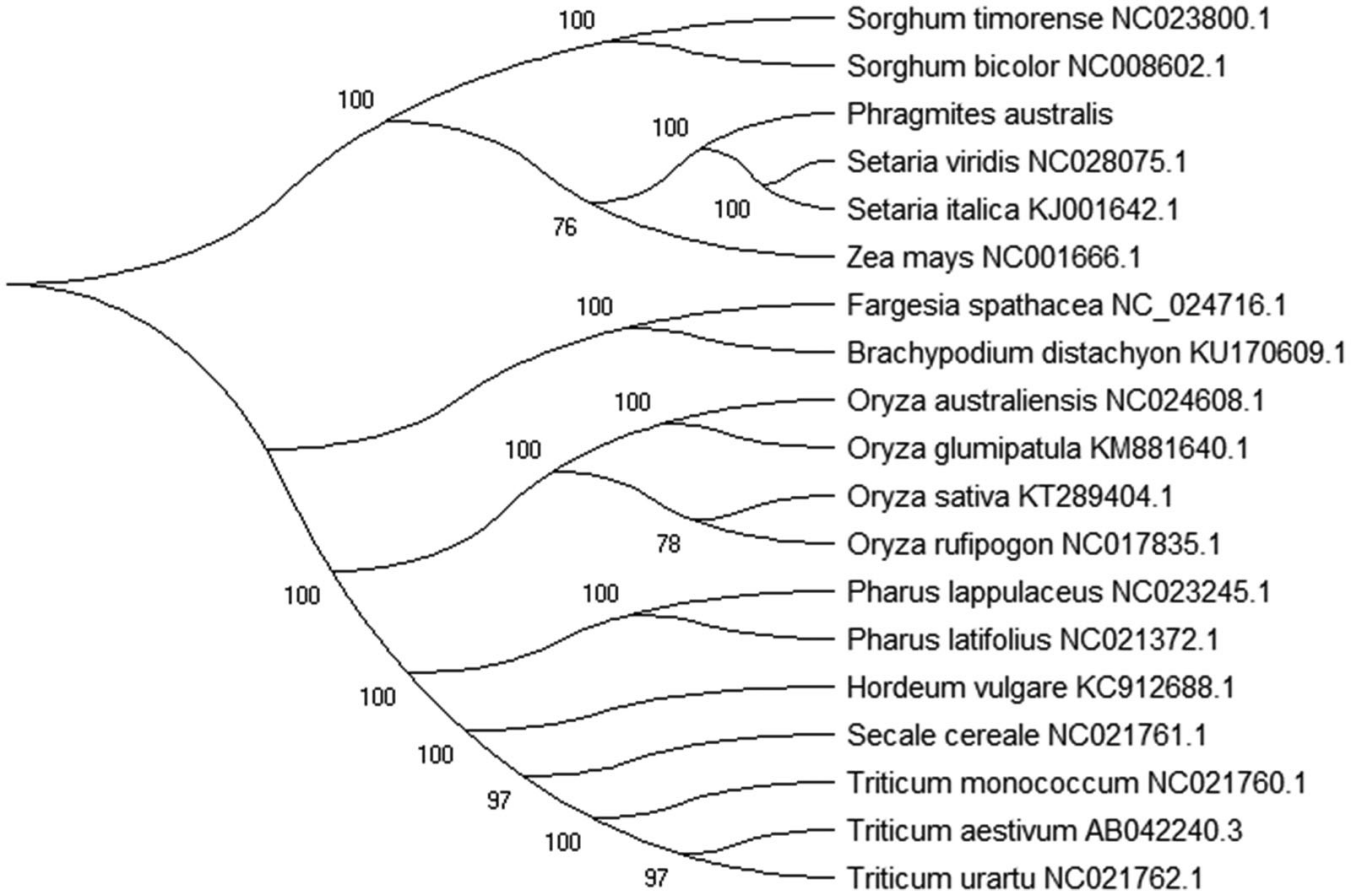
Phylogenetic relationships among the chloroplast genome of *Phragmites australis* with other 18 whole chloroplast genomes of the genus Phragmites. Bootstrap support values are given at the nodes and the bootstrap values from 1000 replicates using the maximum-likelihood (ML) method.
